# Rice MPK17 Plays a Negative Role in the *Xa21*-Mediated Resistance Against *Xanthomonas oryzae* pv. *oryzae*

**DOI:** 10.1186/s12284-022-00590-4

**Published:** 2022-08-03

**Authors:** Zheng Zhu, Tianxingzi Wang, Jinping Lan, Jinjiao Ma, Haiqing Xu, Zexi Yang, Yalu Guo, Yue Chen, Jianshuo Zhang, Shijuan Dou, Ming Yang, Liyun Li, Guozhen Liu

**Affiliations:** 1 College of Life Sciences, Hebei Agricultural University, 2596 Lekai South Street, West Campus, Baoding, 071001 Hebei China; 2 Hebei Key Laboratory of Plant Physiology and Molecular Pathology, Baoding, 071001 China; 3 Research Center for Life Sciences, Hebei North University, Zhangjiakou, 075000 Hebei China; 4 Agricultural Genomics Institute at Shenzhen, Chinese Academy of Agricultural Sciences, Shenzhen, 518116 Guangdong China

**Keywords:** Rice, Bacterial blight, Resistance gene *Xa21*, MAPK, WRKY, Transcription factor

## Abstract

Rice bacterial blight, caused by *Xanthomonas oryzae* pv*. oryzae* (*Xoo*), is one of the most serious diseases affecting rice production worldwide. *Xa21* was the first disease resistance gene cloned in rice, which encodes a receptor kinase and confers broad resistance against *Xoo* stains. Dozens of components in the *Xa21*-mediated pathway have been identified in the past decades, however, the involvement of mitogen-activated protein kinase (MAPK) genes in the pathway has not been well described. To identify MAPK involved in *Xa21*-mediated resistance, the level of MAPK proteins was profiled using Western blot analysis. The abundance of OsMPK17 (MPK17) was found decreased during the rice-*Xoo* interaction in the background of *Xa21*. To investigate the function of MPK17, MPK17-RNAi and over-expression (OX) transgenic lines were generated. The RNAi lines showed an enhanced resistance, while OX lines had impaired resistance against *Xoo*, indicating that MPK17 plays negative role in *Xa21*-mediated resistance. Furthermore, the abundance of transcription factor WRKY62 and pathogenesis-related proteins PR1A were changed in the MPK17 transgenic lines when inoculated with *Xoo*. We also observed that the MPK17-RNAi and -OX rice plants showed altered agronomic traits, indicating that MPK17 also plays roles in the growth and development. On the basis of the current study and published results, we propose a “*Xa21*-MPK17-WRKY62-PR1A” signaling that functions in the *Xa21-*mediated disease resistance pathway. The identification of MPK17 advances our understanding of the mechanism underlying *Xa21*-mediated immunity, specifically in the mid- and late-stages.

**Supplementary Information:**

The online version contains supplementary material available at 10.1186/s12284-022-00590-4.

## Background

Rice (*Oryza sativa* L.) is the stable food for more than half of the world’s population and it is the model organism for plant molecular biology research. Bacterial blight caused by *Xanthomonas oryzae* pv. *oryzae* (*Xoo*) is the most serious bacterial disease of rice and leads to heavy yield losses (Mark et al. 1994; Liu et al. 2014). To fight against attacking pathogens, plants have evolved two layers of defense strategy: pathogen-associated molecular pattern (PAMP)-triggered immunity (PTI) and effector-triggered immunity (ETI) (Jones and Dangl 2006). More than 40 resistance genes to *Xoo* stains have been identified, of which 11 have been characterized at the molecular level in the past decades (Jiang et al. 2020).

*Xa21* was the first resistance gene cloned in rice, which harbors broad spectrum of resistance to *Xoo*, and is widely utilized in rice breeding programs. *Xa21* encodes a receptor-like kinase (RLK) with three typical domains, including a leucine-rich repeat (LRR) domain, a transmembrane domain and an intracellular kinase domain (Song et al. 1995). The LRR domain recognizes the sulfurized RaxX (Required for activating *Xa21*-mediated immunity X) polypeptide from *Xoo*, thereby triggering an intracellular defense response (Pruitt et al. 2015). After years of research, dozens of components have been identified in *Xa21*-mediated signaling, including the E3 ubiquitin ligase XB3, which is a substrate of XA21 and has a role in XA21 stability (Wang et al. 2006). The *Xb10* gene encodes the transcription factor OsWRKY62, which negatively regulates the transcription of downstream pathogenesis-related (PR) genes and reduces the resistance once overexpressed (Peng et al. 2008). OsWRKY62 is located in the nucleus and can interact with the kinase domain of XA21 protein (Park and Ronald 2012). While the phosphatase XB15 can dephosphorylate XA21, the plant-specific ankyrin domain protein XB25 is required for XA21 stability (Park et al. 2008; Jiang et al. 2013). XB21 protein belongs to the accessory protein family and overexpression of the *Xb21* gene in the presence of *Xa21* enhanced rice resistance to *Xoo* stains (Park et al. 2017). ATPase (XB24) binds to XA21 and promotes the phosphorylation of the XA21 protein (Chen et al. 2010). RAR1, WAK25 and SnRK1 were also shown to affect *Xa21*-mediated resistance in rice (Seo et al. 2011). In addition, BIP3, SDF2, SERK2 and XIK1 were identified via yeast-two-hybrid or pull-down approaches (Park et al. 2010a, 2013; Chen et al. 2014; Hu et al. 2015; Jiang et al. 2020; Joshi et al. 2020).

*Xa21* confers full resistance only at the 6-week-old stage or later, but not at the seedling stage (2-week-old) (Mazzola et al.^1994^; Century et al. 1999; Zhao et al. 2009). This makes it difficult to screen plants with enhanced resistance. Recently, It was showed that *Xa21* can confers full resistance at the seedling stage when the plants are grown at lower temperatures, which facilitates the characterization of *Xa21* resistance (Chen et al. 2018).

The highly conserved mitogen-activated protein kinase (MAPK) cascade is composed of three kinds of protein kinases: MAPKKK, MAPKK, and MAPK. Activated MAPKs enter the nucleus and activate specific transcription factors (TF) (Zhang et al. 2018; Jalmi and Sinha 2016). Through an in silico search of the rice genome databases, 75 genes encoding MAPKKK, 8 genes encoding MAPKK and 17 genes encoding MAPK were identified (Reyna and Yang 2006). Accumulated evidence supports that the MAPK cascade plays crucial roles in diverse stress responses including the disease resistance in rice. Transcriptome analysis showed that the transcription levels of 74 MAPKKKs, 8 MAPKKs and 17 MAPKs are altered in two pairs of resistant/susceptibility rice plants after inoculated with *Xoo*, and most of them were up-regulated in incompatible interactions, suggesting that the MAPK genes play an important role in the process of rice-*Xoo* interactions (Yang et al. 2015). Resistance against *Xoo* was enhanced by *OsMPK5* (Os03g17700) knockdown or *OsMPK15* (Os11g17080) and *OsEDR1* (Os03g06410) knockout, indicating that *OsMPK5*, *OsMPK15*, and *OsEDR1* have a negative role in the resistance (Xiong and Yang 2003; Hong et al. 2019; Shen et al. 2011). On the other hand, the overexpression of *OsMPK7* (Os06g48590), *OsMPK12-1* (Os06g49430), *OsMKK3* (Os06g27890) and *OsMPKK6* (Os01g32660) led to an enhanced resistance against *Xoo*, indicating that they play a positive role in the resistance responses (Xiao et al. 2017; Jalmi and Sinha 2016; Li et al. 2021). Shen et al. demonstrated that *OsMPK6* (Os10g38950) plays dual functions as an activator and an inhibitor in the immune pathway (Shen et al. 2010). Although a number of MAPKs appear to be involved in the rice-*Xoo* interactions, no direct experimental data have been obtained to demonstrate the function of MAPK genes in *Xa21-*mediated immunity.

Currently, most of the components in the *Xa21*-mediated disease resistance pathway were identified by yeast-two-hybrid or pull-down assays (Jiang et al. 2020). To identify components that do not directly bind to XA21, antibody-based rice proteomics (AbRP) strategy was proposed to identify candidate genes based on the alteration of protein abundance (Liu et al. 2011). Using the AbRP approach, a number of elements might be involved in the *Xa21*-mediated pathway have been identified in our laboratory, including transcription factor and pathogenesis-related (PR) genes (Miao et al. 2014; Yang et al. 2016; Wu et al. 2011; Hou et al. 2012). Further experiments by RNA interference (RNAi) or overexpression demonstrated that WRKY42 and WRKY68 are positively involved in *Xa21*-mediated immunity (Wang et al. 2021; Zhu et al. 2021).

We reported previously that overexpressing MPK17 enhances drought tolerance in rice (Ma et al. 2019). To explore the functions of MPK17 in rice-*Xoo* interactions, MPK17-RNAi and MPK17-OX transgenic plants were generated in the background of *Xa21,* our data suggest that MPK17 plays a negative role in *Xa21*-mediated immunity.

## Materials and Methods

### Plants, Pathogen Strains and Antibodies

Rice variety TP309 (*Oryza sativa*. Sub. Japonica), which is susceptible to bacterial leaf blight (*Xanthomonas oryzae* pv. *oryzae*, *Xoo*) strain PXO99 (compatible response, S) and 4021, which is a homozygous strain of TP309 harboring *Xa21*, resistant to Philippine race 6 PXO99 (incompatible response, R) (Xu et al. 2006) were used in this study. The rice plants were cultivated in the greenhouse and experimental field located at the West campus of Hebei Agricultural University (Baoding, China).

Anti-*HSP82* (Os09g30418) monoclonal antibody (Li et al. 2011), anti-*MPK17* (Os05g50120) monoclonal antibody (Ma et al. 2019), anti-*Xoo* polyclonal antibody (Guo et al. 2015), anti-*WRKY42* (Os02g26430) (Miao et al. 2014), anti-*WRKY68* (Os04g51560) (Yang et al. 2016), anti-*WRKY62* (Os09g25070), anti-*WRKY67* (Os05g09020), anti-*WRKY76* (Os09g25060) and anti-*PR1a* (Os07g03710) polyclonal antibodies (Wu et al. 2011), horseradish peroxidase-labeled goat anti-rabbit polyclonal antibody and goat anti-mouse monoclonal antibodies were purchased from Beijing Protein Innovation Co., Ltd., Beijing, China.

### MPK17-RNAi and OX Vector Construction

A plasmid containing the *MPK17* cDNA, purchased from the Rice Genome Resource Center of the Japan Agricultural Bioresources Research Institute was used as template for PCR amplification. A fragment of 200 bp MPK17 cDNA was selected to construct the MPK17-RNAi vector. The RNAi target sequence is located at 241–495 nt of MPK17 cDNA, the upstream primer sequence is 5’-GCGGATCCGAGCTCTTGGCCAAAGATGATGTCCG-3’, the downstream primer sequence is 5’-GGGGTACCACTAGTCCAATTGCTCTTTCCCTTGG-3’. The underlined sequences were restriction enzyme recognition sites. The RNA interference fragment was inserted into recombinant plasmid pEASYT1-Intron, and verified by double digestion with *Hind* III and *Sac* I. After validation by sequencing, the RNA interference fragment was released, purified and ligated with transformation vector pCAMBIA2300 to generate pCAMBIA2300-MPK17-RNAi, which was further verified by *Hind* III and *Pst* I digestion and then used to transform rice 4021 (Zhu et al. 2021). The detailed protocols for constructing MPK17-OX were described previously (Ma et al. 2019).

### Rice Transformation and the Identification of Transgenic Plants

Rice 4021 was transformed via Agrobacterium-mediated method at Boyuan Biotechnology Co., Ltd., Wuhan, China, according to the protocol (Kaur et al. 2016). The upstream primer for PCR identification of MPK17-RNAi transgenic lines was 5’-GAGTCGTAAGAGACTCTGTATG-3’, which is located in the 35S promoter region. The downstream primer was 5’- GCGAGCTCGGTTTTCAGTTGAGCAAC-3’, which is located in the intron part of the RNAi vector. The upstream primer for PCR used to identify MPK17-OX transgenic lines was 5’-GCGGTACCATGGGCGGCCGCGCCCGCTC-3’, the downstream primer was 5’-GCGAGCTCGGTTTTCAGTTGAGCAAC-3’. PCR amplifications were carried out using the follow cycling parameters: pre-denaturation at 94 °C for 3 min, followed by 35 cycles of denaturation at 94 °C for 30 s, annealing at 56 °C for 30 s, and extension at 72 °C for 1 min, with a final extension at 72 °C for 10 min. The PCR products were detected by electrophoresis using 1.0% agarose gel.

### Rice Cultivation and Agronomic Traits Investigation

Rice plants were grown in a plant growth chamber (30℃, humidity 50–60%, 15 h light/9 h dark cycle, light intensity 4100 Lux), rice seedlings at three-leaf stage were used for inoculation with *Xoo* (Zhu et al. 2021). Rice plants grown in an experimental field were used to investigate the phenotype and photographed at different growth and development stages. Plant height, number of grains per spike, panicle length, seed setting rate and 1000-grain weight of at least 8 individual plants for each transgenic line were measured at the maturity stage. The mean and standard deviation were calculated and then statistics analysis was carried out.

### *Xoo* Culture and Rice Inoculation

*Xoo* was cultured on PDA (potato 200 g·L^−1^, glucose 15 g·L^−1^ and agar 15 g·L^−1^) medium at 28℃ for 2–3 days. Bacterial cells were collected and suspended in sterile distilled water, and the optical density was adjusted to 0.5 at 600 nm detected by a spectrophotometer (Hou et al. 2012). The rice seedlings at three-leaf stage were inoculated with the *Xoo* suspension. The tips (about 2 cm) of the upmost leaf were cut off using a sterilized scissor which had been dipped into the bacterial solution prior to cutting. After inoculation, the rice seedlings were cultivated in the incubator at 27℃ or 31℃ with humidity 50–60% and 15 h light/9 h dark cycle (Chen et al. 2018).

### Lesion Measurement and Sample Collection

The lengths of lesion in 8 inoculated leaves were measured daily after inoculation. The means and standard deviation of the length were calculated for statistical analysis. The two representative leaves of each line were pictured at 12th day after inoculation. The portion of leaves (± 1 cm of lesion) were collected, and snap-frozen in liquid nitrogen and stored at −80 °C (Zhu et al. 2021).

### Total Protein Isolation and Western Blot (WB) Analysis

The rice leaves were grinded into powder using a high-throughput shaking mill chilled with liquid nitrogen. One ml extraction buffer (62.5 mmol·L^−1^ Tris–HCl, pH 7.4, 10% glycerol, 2% SDS, 1 mmol·L^−1^ PMSF, 2 mmol·L^−1^ EDTA, 100 mmol·L^−1^ DTT) was added to 0.1 g sample. The supernatants were collected by centrifugation at 12,000×*g* at 4 °C for 20 min, added with one forth volume of loading buffer (50 mmol·L^−1^ Tris, pH 6.8, 200 mmol·L^−1^ DTT, 2% SDS, 0.1% bromophenol blue, 10% glycerol). The proteins in the supernatants were denatured by heating in boiling water for 20 min and were stored at -20℃ till use (Li et al. 2011).

Total protein was separated on 10% SDS-PAGE or tricine gel (160 V, 80 min) according to the molecular weight of the proteins, then transferred to PVDF membrane (100 V, 60 min). After the membrane was incubated with antibodies, super-sensitivity ECL luminescent solution (Baizhi Bio, Beijing, China) was dropped onto the membrane to produce signals. The intensity of signal was detected by a chemiluminescence imager (Beijing Saizhi Venture Technology Co., Ltd., MiniChemi610) (Bai et al. 2012). The band detected by anti-*HSP82* antibody was used as loading control (Li et al. 2011). Protein abundance was measured using Lane 1D software (Baizhi Bio, Beijing, China).

### Statistical Analysis

Data were analyzed and plotted using Windows Microsoft Excel 2010 (Microsoft, London, UK). Means from triplicate experiments were presented with error bars showing standard deviations. Differences among treatments were analyzed, *p* < 0.05 and *p* < 0.01 were considered statistically significant and extremely significant, respectively.

## Results

### MPK17 was Down-Regulated Following *Xoo* Inoculation

**Fig. 1:**
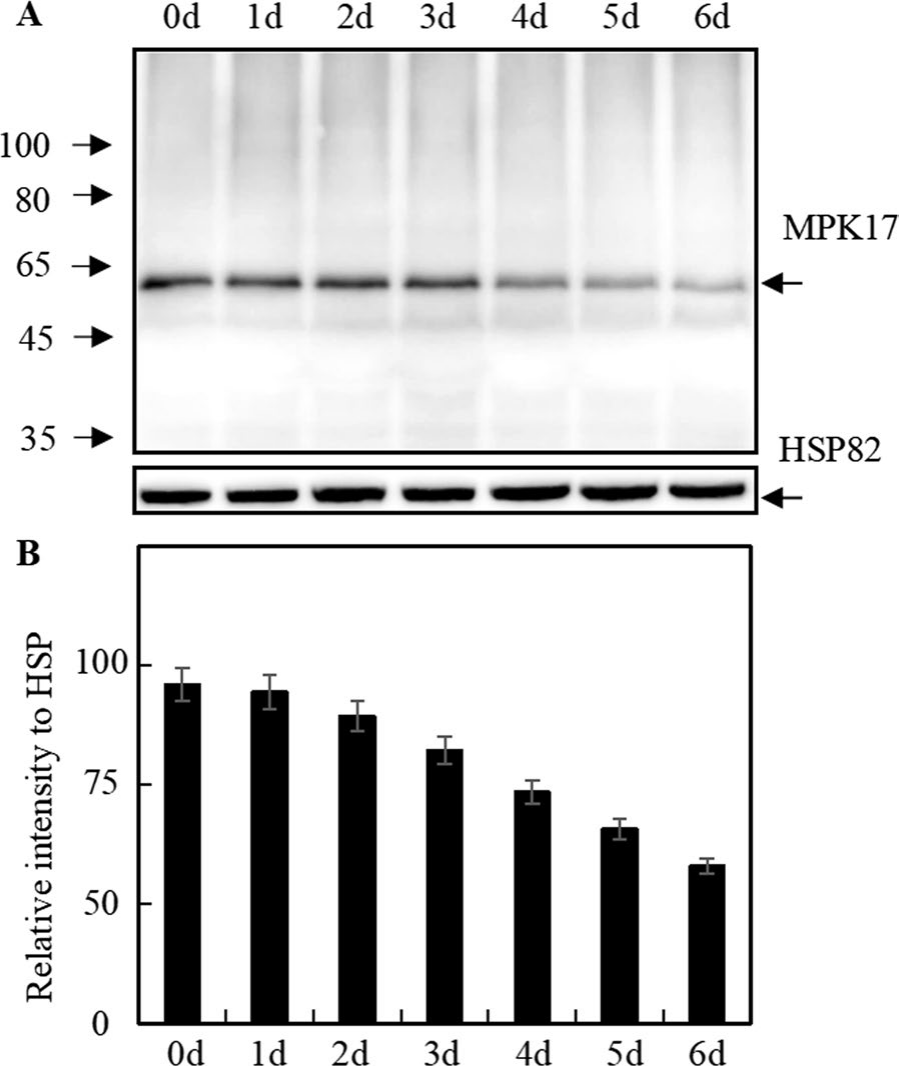
Expression of MPK17 in rice leaves at different time points following inoculation with *Xoo*. **A** Representative Western blot. **B** Expression level relative to HSP82. MPK17: anti-*MPK17* antibody-detected band; HSP82: anti-*HSP82* antibody detected-band used as loading control. 4021: transgenic TP309 lines harboring the *Xa21* gene. Protein signals generated by WB were detected by Mini Chemiluminescent Imager and Sage Capture software. Lane 1D software was used to extract signals of WB. Relative intensity to HSP82 were calculated according to the intensity of MPK17 divided by the intensity of HSP82 signal. Mean was calculated for three repeats. Error bars are standard deviation (SD)

We generated antibody against MPK17 using purified MPK17 protein as immunogen and the specificity was verified by transgenic rice plants overexpression of MPK17. It was found that the abundance of MPK17 protein was up-regulated under drought stress and down-regulated under abscisic acid and methylene jasmonate acid treatments (Ma et al. 2019). To detect the abundance of MPK17 during rice-*Xoo* interaction, rice leaf samples from 4021 were collected at different time points after inoculation, and WB analysis was performed (Fig. 1). As shown in Fig. 1, the protein levels of MPK17 were substantially decreased to 46.0% after *Xoo* inoculation when observed at 6 dpi, suggested that *MPK17* gene is participated in *Xa21*-mediated resistance to bacterial blight. In contrast, the protein levels of MPK17 were stable in TP309 plants after inoculation of *Xoo* (Additional file 1: Fig. S1A). Additionally, no significant difference were detected for the abundance of XA21 protein in MPK17-RNAi and MPK17-OX plants (Additional file 1: Fig. S1B).

### MPK17 Changed Agronomic Traits of Transgenic Plants

In order to explore the function of the *MPK17* gene, RNA interference (RNAi) and overexpression experiments were performed. Construction of the transformation plasmids was illustrated in Additional file 1: Fig. S2. *Agrobacterium*-mediated method was used to transform the rice line 4021, which harbors the *Xa21* gene.

**Fig. 2:**
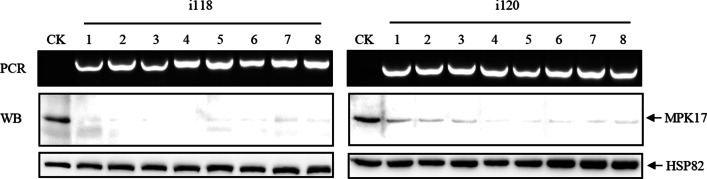
Identification of MPK17-RNAi transgenic lines by PCR and WB. i118, i120: independent MPK17-RNAi transgenic lines; CK: 4021 (homozygous TP309 transgenic line harboring the *Xa21* gene); 1–8: individual transgenic plants in T_3_ generation; PCR: PCR genotyping results done with primers (5’-GAGTCGTAAGAGACTCTGTATG-3’ and 5’- GCGAGCTCGGTTTTCAGTTGAGCAAC-3’). MPK17: anti-MPK17 antibody-detected band; HSP82: anti-HSP82 antibody-detected band used as loading control. PCR: PCR product separated on agarose gel. WB: detection of MPK17 using anti-MPK17 antibody

Nineteen independent MPK17-RNAi lines were obtained in T_0_ generation. PCR positive plants were selected, independent and homozygous transgenic lines were identified in the subsequent generations. WB analysis revealed that the intensity of MPK17 was drastically reduced in the homozygous MPK17-RNAi lines i118 and i120 (Fig. 2, Additional file 1: Fig. S3A). The trancriptional abundance of MPK17 gene were detected by reverse transcription PCR (RT-PCR), and the result showed that the transcriptional signal of MPK17 gene in RNAi line were about one fifth of TP3309 and 4021controls (Additional file 1: Fig. S5).

**Fig. 3:**
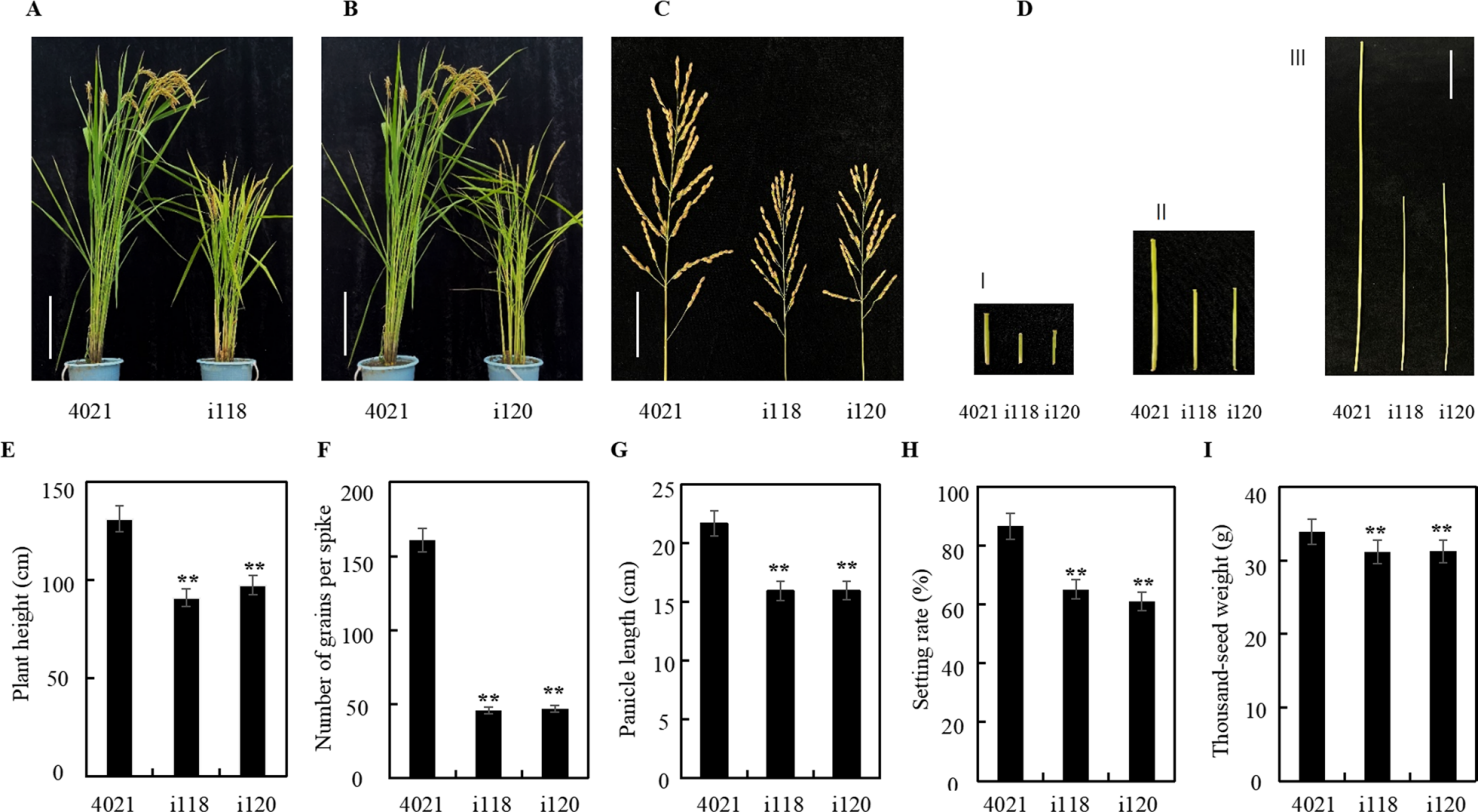
Agronomic traits of MPK17-RNAi transgenic plants. **A** and **B** 4021 control and MPK17-RNAi transgenic lines at mature stage (bar = 30 cm). **C** Panicles of 4021 and MPK17-RNAi transgenic lines at mature stage (Bar = 5 cm). **D** Internodes of 4021 and MPK17-RNAi transgenic lines at mature stage (Bar = 10 cm). I: the third internode from the top; II: the second internode from the top; III: the upmost internode. **E** Columns showing plant height. **F** Columns showing number of grains per spike. **G** Columns showing for panicle length. **H** Columns showing seed setting rate. **I** Columns showing 1000-grain weight. i118 and i120: independent MPK17-RNAi transgenic lines; 4021: TP309 transgenic line harboring the *Xa21* gene. Mean was calculated for eight individual plants. Error bars are standard deviation (SD). “**” designate difference at *p* < 0.05 level

The agronomic traits were assessed at the mature stage (Fig. 3). The plant height of the two MPK17-RNAi lines were 91.0 cm and 97.5 cm, respectively, shorter than 4021 plant (131.3 cm). Compared with 4021, the internodes length, the number of grains per spike, panicle length, seed setting rate and 1000-grain weight of the MPK17-RNAi transgenic plants were decreased significantly, indicating that the down-regulation of MPK17 changed the morphology of rice plant in multiple aspects and MPK17 plays important roles for rice growth and development.

**Fig. 4:**
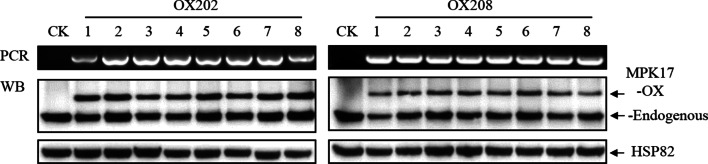
Identification of MPK17-OX transgenic lines by PCR and WB. OX202 and OX208: independent MPK17-OX transgenic lines; CK: 4021 (homozygous TP309 transgenic line harboring the *Xa21* gene); 1–8: individual transgenic plants in T_3_ generation; PCR: PCR genotyping results done with primers (5’-GCGGTACCATGGGCGGCCGCGCCCGCTC-3’, 5’-GCGAGCTCGGTTTTCAGTTGAGCAAC-3’). MPK17-OX: the transgenic MPK17 protein; MPK17-Endogenous: the endogenous MPK17 protein in rice; HSP82: anti-HSP82 antibody-detected band used as loading control

Twenty-five independent MPK17-OX transgenic lines were obtained in T_0_ generation. PCR and/or WB were used to select positive and homozygous lines. Independent and homozygous lines OX202 and OX208 (MPK17-OX transgenic) were obtained in T_3_ generation (Fig. 4, Additional file 1: Fig. S3B). As 3 × HA tags was integrated into the MPK17-OX version, the molecular weight is larger than that the endogenous MPK17, thus two bands were detected in the overexpression lines by WB. The band with molecular weight about 75 kD was verified by WB using anti-HA antibody (Additional file 1: Figs. S4A, S4B). The band with smaller molecular weight (about 66 kD) was the endogenous rice MPK17 protein (MPK17-Endogenous). It can be seen that the intensity of the two bands were pretty close, indicated that the abundance of over-expressed MPK17 and the endogenous one is similar. RT-PCR analysis showed that the transcriptional abundance of MPK17 gene in over expression lines were about 2.8 times higher than that in TP309 and 4021 controls (Additional file 1: Fig. S5).

**Fig. 5:**
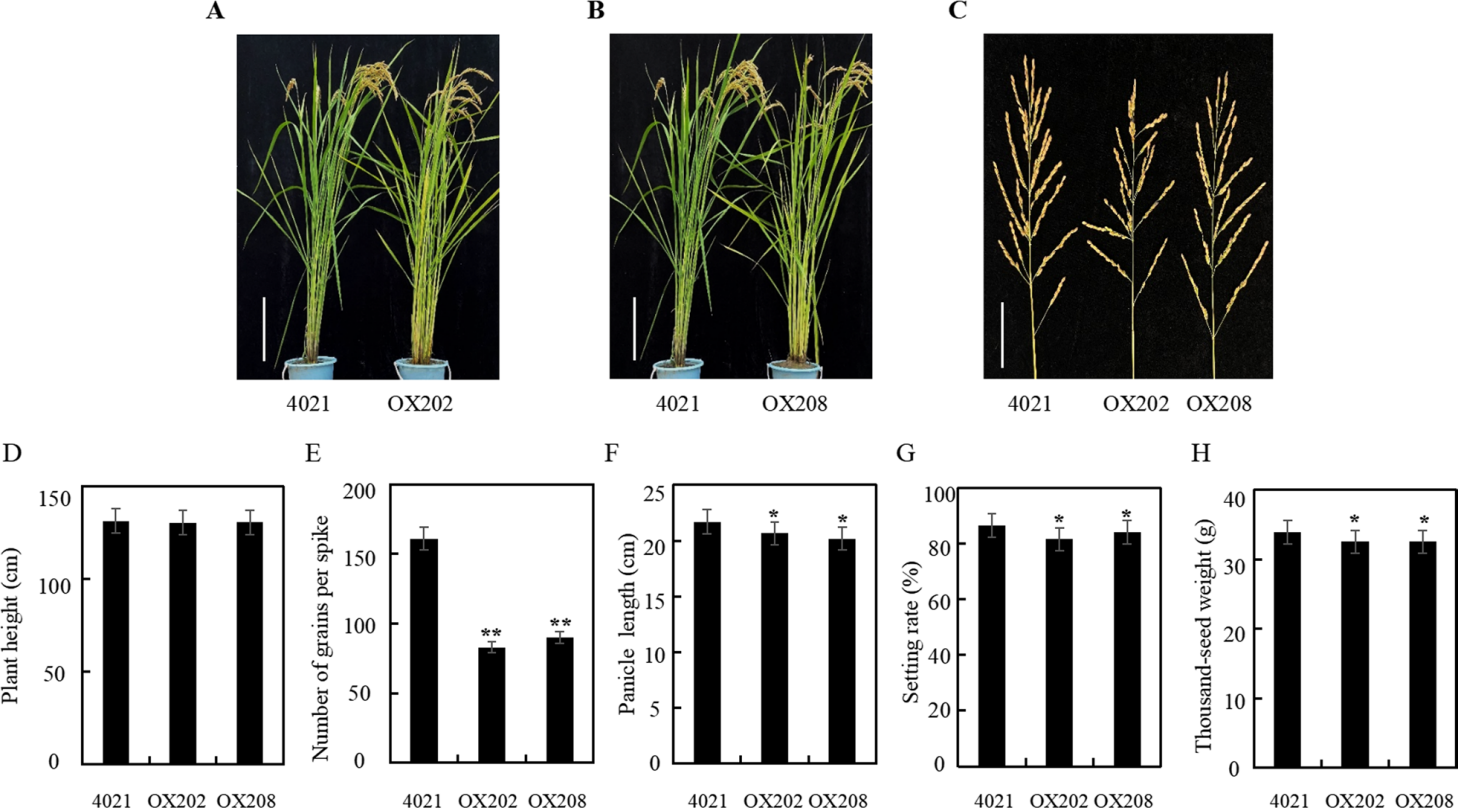
Agronomic characters of MPK17-OX transgenic plants. **A**, **B** 4021 and MPK17-OX transgenic lines at mature stage (Bar = 30 cm). **C** Panicles of 4021 and MPK17-OX transgenic lines at mature stage (Bar = 5 cm). **D** Columns showing plant height. **E** Columns showing numbers of grains per spike. **F** Columns showing for panicle length. **G** Columns showing for seed setting rate. **H** Columns showing 1000-grain weight. OX202 and OX208: independent MPK17-OX transgenic lines; CK: 4021 (TP309 transgenic line harboring the *Xa21* gene). Mean was calculated for eight individual plants. Error bars are standard deviation (SD). “*” and “**” designate difference at *p* < 0.05 and *p* < 0.01 levels respectively

Compared with the 4021 control, the plant height of MPK17-OX transgenic rice was similar, while the number of grains per spike, panicle length, seed setting rate and 1000-grain weight were decreased significantly (Fig. 5). The phenotypic changes were similar with our previous report using TP309 as transgenic recipient (Ma et al. 2019), suggesting that morphology alteration is independent of *Xa21* gene. The phenotypic changes derived from RNAi or overexpression of MPK17 revealed that certain level of MPK17 is necessary for normal growth of rice, while high level of MPK17 has less impact on rice.

### Down-Regulation of MPK17 Enhanced *Xa21*-Mediated Resistance Response

**Fig. 6:**
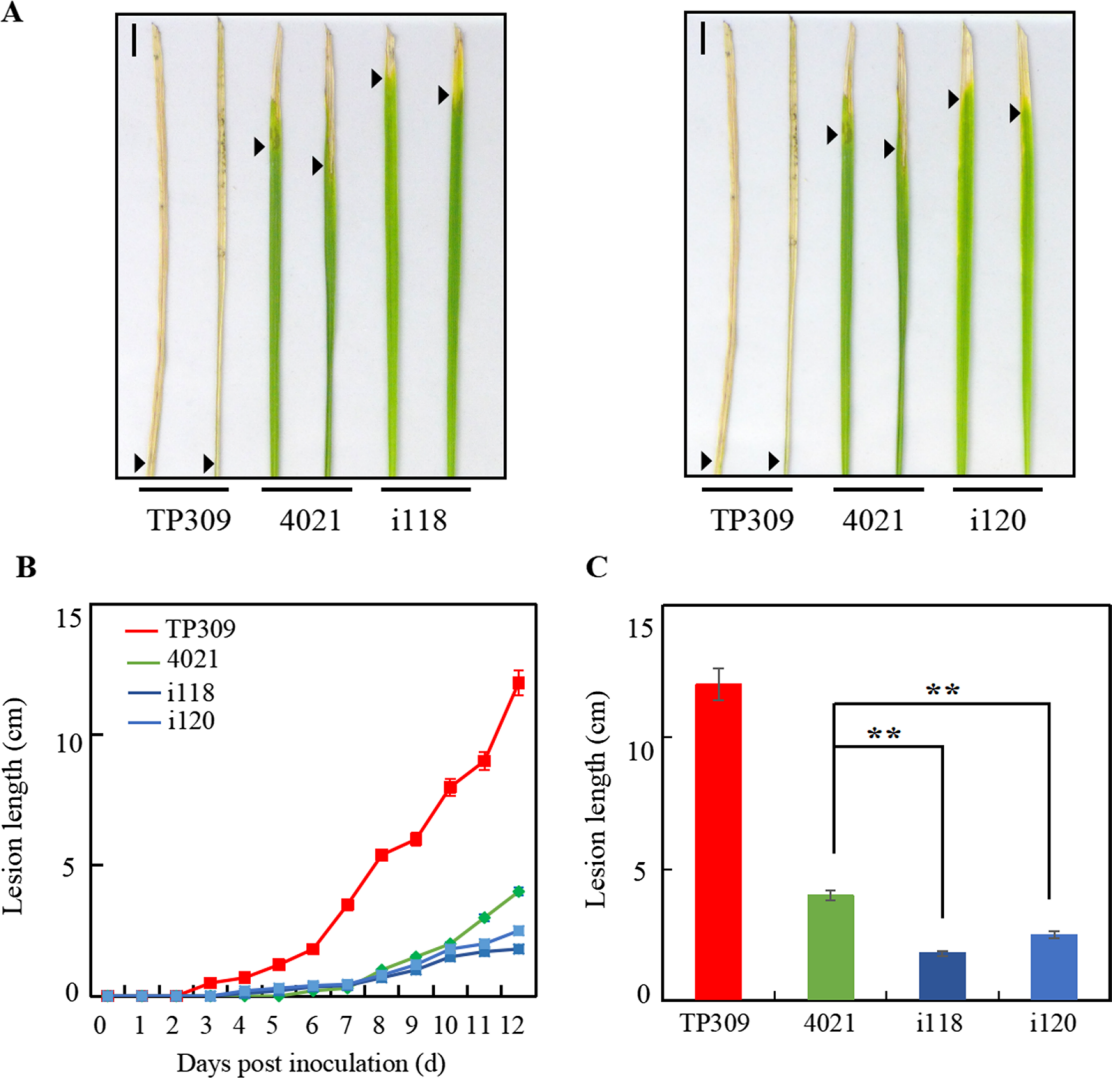
MPK17 knockdown enhanced *Xa21*-mediated resistance. **A** Disease lesions in inoculated leaves of i118 and i120 and control plants at 12 dpi. **B** Lesion length at different dpi. **C** Lesion length at 12 dpi. Arrows indicate the frontline of lesions. **denotes difference at *p* < 0.01 level. Bar = 1 cm. i118 and i120: independent MPK17-RNAi transgenic lines; TP309: Japonica rice; 4021: homozygous TP309 transgenic line harboring the *Xa21* gene

Chen et al. (2018) reported that 4021 showed impaired resistance to *Xoo* strains at 31 °C, to test the resistance response for MPK17 down-regulated plants, MPK17-RNAi transgenic plants of i118 and i120 were inoculated and grown at 27 or 31 °C and lesion length was subsequently measured. At 31 °C condition, the lesion in TP309 appeared at 3 dpi, while the lesion in the 4021 and MPK17-RNAi transgenic plants appeared at 5 or 6 dpi. The expansion of lesions was faster in TP309 than in 4021 and transgenic plants. At 12 dpi, the lesion covered most of the leaves in TP309 with a mean lesion length of 12.0 cm, while the lesion length in 4021 was 4.0 cm, and 1.8 cm and 2.5 cm in i118 and i120, respectively (Fig. 6A, B). Statistical analysis showed that there were significant differences in the lesion length among the three groups, while no significant differences between the two transgenic lines (Fig. 6C). At 27 °C condition, compared with 4021 control, no significant differences were detected in MPK17-RNAi transgenic plants as both 4021 and MPK17-RNAi showed full resistance responses. This result indicated that the down-regulation of MPK17 enhances *Xa21*-mediated resistance to *Xoo*.

### MPK17 Overexpression Impaired the *Xa21*-Mediated Resistance

**Fig. 7:**
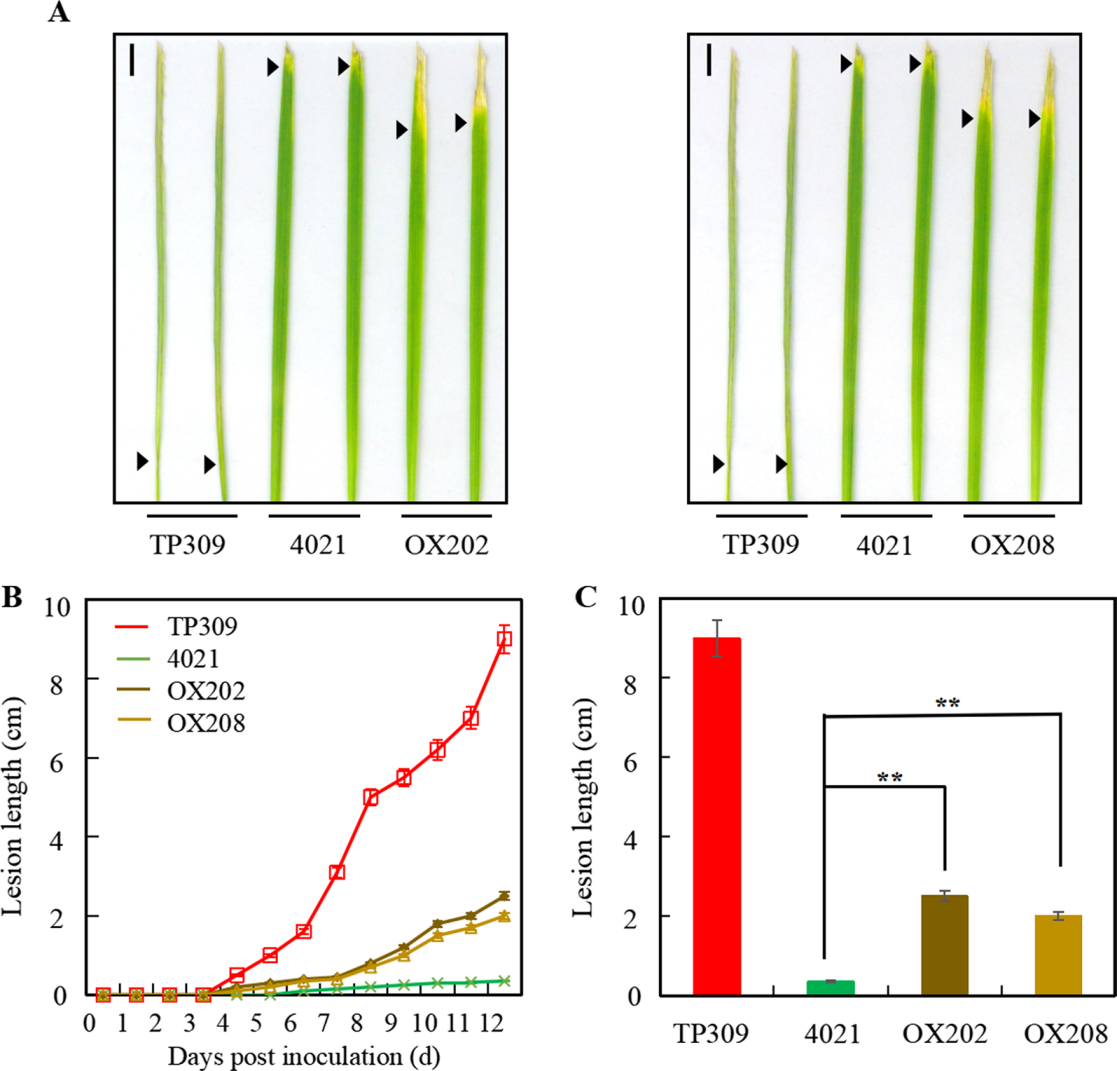
*Xa21*-mediated resistance in rice following overexpression of MPK17 at 27 °C. **A** Lesions in inoculated leaves of OX202, OX208 and control plants at 12 dpi. **B** Lesion length at different dpi. **C** Lesion length at 12 dpi. Arrows indicate the frontline of lesions. **designate difference at *p* < 0.01 level. Bar = 1 cm. OX202 and OX208: independent MPK17-OX transgenic lines; TP309: Japonica rice variety; 4021: TP309 transgenic line harboring the *Xa21* gene

MPK17-OX lines OX202 and OX208 were also inoculated and grown at 27 or 31 °C to assess lesion development. At 27 °C condition, the lesions in TP309 appeared at 4 dpi, while the lesions in 4021, OX202 and OX208 appeared at 6 or 7 dpi. The expansion of lesions was faster in TP309 than in 4021 and MPK17-OX plants. At 12 dpi, the mean lesion lengths were 9.0 cm in TP309, 0.35 cm in 4021, but 2.5 cm and 1.8 cm in OX202 and OX208, respectively, and were significantly different (*p* < 0.01) among TP309, 4021 and transgenic lines (Fig. 7A–C). The increased lesions in OX202 and OX208 as compared to 4021 indicated that *Xa21*-mediated resistance to *Xoo* strain was impaired when MPK17 was overexpressed. At 31 °C condition, the expansion of lesions in MPK17-OX was faster than that in 4021 plants and at 12 dpi, the mean lesion lengths in MPK17-OX plants were longer than 4021 (*p* < 0.05) (Additional file 1: Fig. S5). These results are consistent with above results obtained in RNAi experiments. Taken together, our data demonstrated that the *MPK17* gene plays a negative role in *Xa21*-mediated resistance.

### The Effect of MPK17 Abundance to the Propagation of *Xoo* in Rice Leaves

In order to explore the effects of MPK17 abundance on the propagation of *Xoo* in rice leaves, the whole leaves were harvested at 0, 6 and 9 dpi, and total protein was extracted and *Xoo* proteins were detected by WB using anti-*Xoo* polyclonal antibodies (Additional file 1: Fig. S6) and signals were used for quantification of *Xoo* as previously (Guo et al. 2015).

**Fig. 8:**
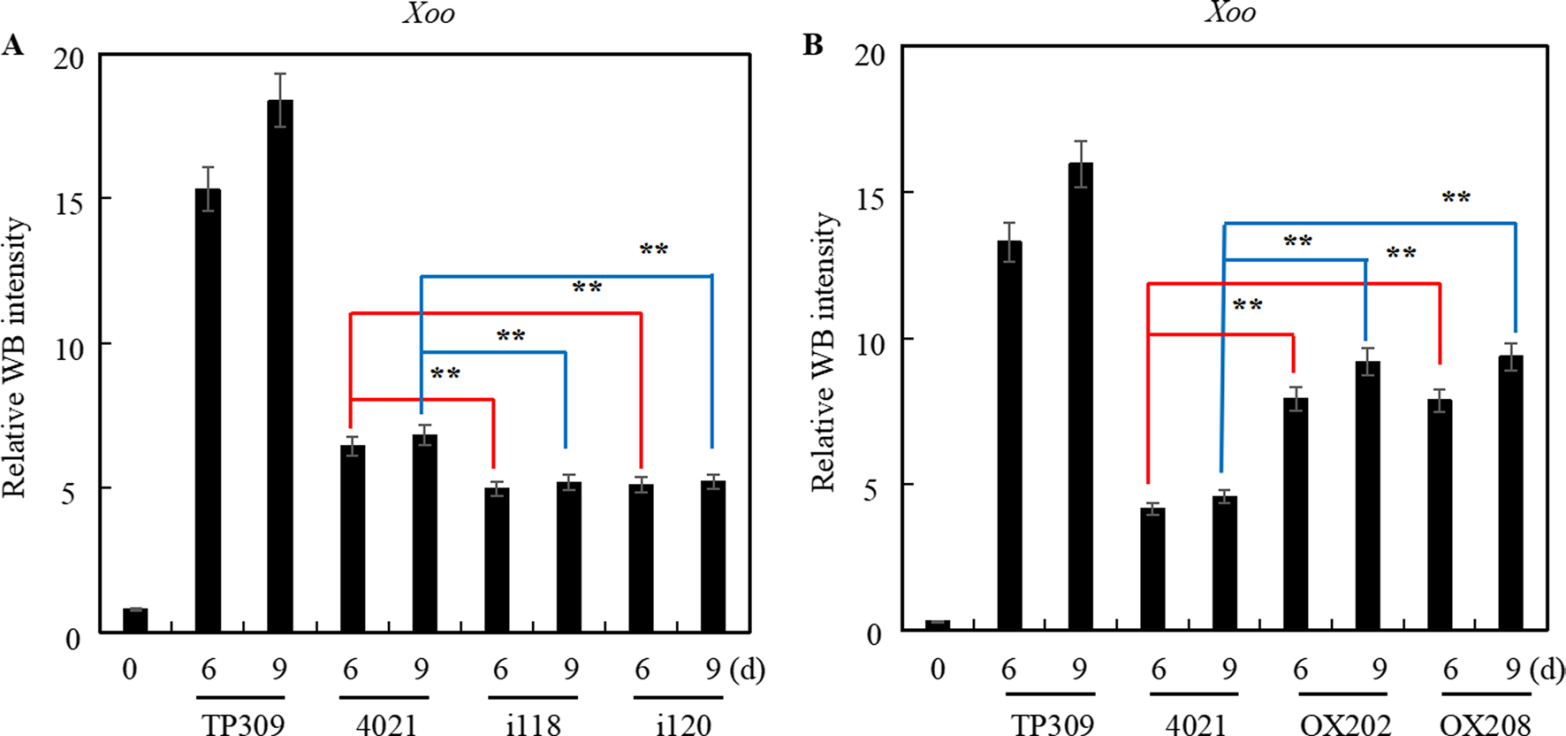
Abundance of *Xoo* proteins in rice expressing different levels of MPK17 following *Xoo* inoculation. **A** MPK17-RNAi transgenic plants. **B** MPK17-OX transgenic plants. Inoculated rice plants were cultivated at 31℃ (MPK17-RNAi transgenic rice) or 27℃ (MPK17-OX transgenic rice). The whole leaves were collected at 0, 6, 9 dpi, total proteins were isolated and separated by SDS-PAGE, then detected by anti-*Xoo* antibody (Additional file 1: Fig. S2). Protein signal were detected by Mini Chemiluminescent Imager and Sage Capture software. Lane 1D software was used to capture the signals of WB. Mean was calculated for three repeats. Error bars are standard deviation (SD). **denotes difference at *p* < 0.01 level. i118, i120: independent MPK17-RNAi transgenic lines; OX202, OX208: independent MPK17-OX transgenic lines; TP309: Japonica rice variety; 4021: homozygous transgenic TP309 line harboring the *Xa21* gene

Compared with 4021, the abundance of *Xoo* proteins in i118 and i120 was lower (Fig. 8A), implying that the propagation of *Xoo* is prohibited when MPK17 is down-regulated. This result is consistent with the enhanced resistance responses in these lines (Fig. 6). On the other side, the abundance of *Xoo* proteins was increased in OX202 and OX208 plants as compared with 4021 (Fig. 8B), indicating that higher levels of MPK17 favors *Xoo* proliferation, which is consistent with the impaired resistance responses in these lines (Fig. 7). As expected, *Xoo* proteins in the susceptible control TP309 was the highest among the three group samples.

### MPK17 Regulates WRKY62 Expression

**Fig. 9:**
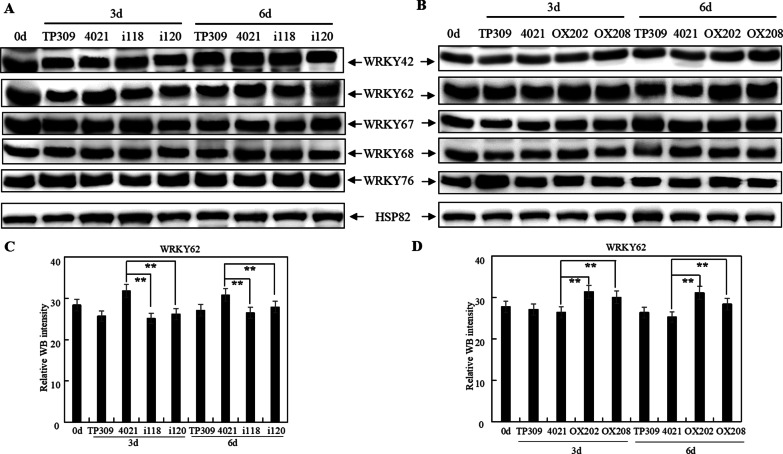
Expression of WRKY in MPK17-regulated transgenic rice after inoculated with *Xoo*. **A** Expression of five WRKY proteins in MPK17-RNAi transgenic rice after inoculated with *Xoo*. **B** Expression of five WRKY proteins in MPK17-OX transgenic rice after inoculated with *Xoo*. **C** Quantitative comparison of WB intensities of WRKY62 protein in panel (**A**). **D** Quantitative comparison of WB intensities of WRKY62 protein in panel (**B**). WRKY42: anti-*WRKY42* antibody-detected band; WRKY62: anti-*WRKY62* antibody-detected band; WRKY67: anti-*WRKY67* antibody-detected band; WRKY68: anti-*WRKY68* antibody-detected band; WRKY76: anti-*WRKY76* antibody-detected band; HSP82: anti-*HSP82* antibody-detected band used as loading control. i118, i120: independent MPK17-RNAi transgenic lines; OX202, OX208: independent MPK17-OX transgenic lines; TP309: Japonica rice variety; 4021: transgenic TP309 line harboring the *Xa21* gene. Signal of WRKY62 protein in panel A and B were detected by Mini Chemiluminescent Imager and Sage Capture software. Lane 1D software was used to extract signals of WB. Average was calculated for three repeats. Error bars are standard deviation (SD). **designate difference at *p* < 0.01 level

In order to identify the elements functioning downstream of MPK17, the expression levels of five reported WRKY transcription factors WRKY42 (Wang et al. 2021), WRKY62 (Peng et al. 2008), WRKY67 (Vo et al. 2017), WRKY68 (Zhu et al. 2021) and WRKY76 (Seo et al. 2011) were monitored using WB (Fig. 9, Additional file 1: Fig. S7). The abundance of WRKY62 was decreased in MPK17 RNAi plants, but increased in MPK17-OX plants at 3 and 6 dpi (Fig. 9A and B). WRKY62 levels were compared quantitatively using captured WB signals, it was found that the abundance of WRKY62 was significantly different between 4021 and the transgenic plants at 3 and 6 dpi (Fig. 9C and D), indicating the abundance of MPK17 and WRKY62 is correlated positively. Meanwhile the abundance of WRKY42, WRKY67, WRKY68 and WRKY76 was similar in MPK17-regulated (MPK17-RNAi and MPK17-OX) transgenic plants and 4021 (Fig. 9A and B). This result suggested that WRKY62 may be regulated by the *MPK17* gene in the *Xa21*-mediated resistance to *Xoo*.

### MPK 17 Affected PR1A Expression

Since PR is associated with disease resistance, we then determined PR protein accumulation in the generated transgenic plants. We investigated the expression of PR1A at different time points after *Xoo* inoculation (Wu et al. 2011) and also found that overexpression of PR1A enhanced *Xa21*-mediated resistance (Liu et al. 2021).

**Fig. 10:**
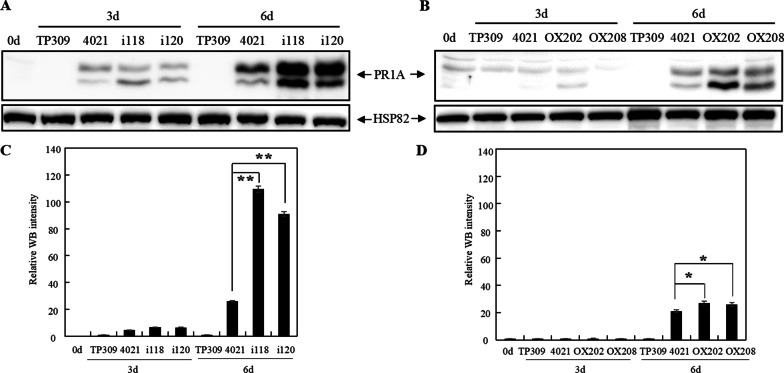
Expression of PR1A in MPK17-regulated transgenic plants following *Xoo* inoculation. **A** Expression of PR1A protein in MPK17-RNAi transgenic rice after inoculated with *Xoo*. **B** Expression of PR1A protein in MPK17-OX transgenic rice after inoculated with *Xoo*. **C** Quantitative comparison of WB intensities of WRKY62 protein in panel (**A**). **D** Quantitative comparison of WB intensities of WRKY62 protein in panel (**B**). PR1A: anti-PR1A antibody detected band; HSP82: anti-HSP82 antibody detected band used as loading control. i118, i120: independent MPK17-RNAi transgenic lines; OX202, OX208: independent MPK17-OX transgenic lines; TP309: Japonica rice variety; 4021: transgenic TP309 line harboring the *Xa21* gene. Signal of PR1A protein in panel (**A**) and (**B**) were detected by Mini Chemiluminescent Imager and Sage Capture software. Lane 1D software was used to extract signals of WB. Average was calculated for three repeats. Error bars are standard deviation (SD). * and **designate difference at *p* < 0.05 and *p* < 0.01 levels respectively

Compared with 4021, PR1A levels in the inoculated leaves were increased extremely significant (*p* < 0.01) in MPK17 RNAi plants (Fig. 10A and C, Additional file 1: Fig. S8A). Interestingly, PR1A levels were also higher significantly (*p* < 0.05) in MPK17 overexpressing plants (Fig. 10B and D, Additional file 1: Fig. S8B). These data supported that PR1A might be regulated by MPK17. In addition, the results showed that the expression of PR1A is also associated with time post *Xoo* inoculation or the number of *Xoo* cells and the existence of *Xa21* gene. The final abundance of PR1A is the comprehensive result of several factors and the abundance change of PR1A alone may not related with the resistance phenotype directly.

## Discussion

We have generated transgenic rice plants in the *Xa21* background with altered expression of MPK17 and *Xa21*-mediated resistance against *Xoo* is either enhanced or compromised. Furthermore, WRKY62 and PR1A levels were altered in the MPK17-regulated transgenic plants, suggesting that these genes may function downstream the *MPK17* gene in *Xa21*-mediated signaling.

### *MPK17 *is a Novel Component in the *Xa21*-Mediated Immunity

Till now, no *MAPK* gene has been identified in the *Xa21*-mediated pathways. Transcriptomic investigation revealed that the transcription level of a number of *MAPK* genes changes as a result of rice-*Xoo* interactions (Yang et al. 2015). In this study, the *Xa21*-mediated resistance response was enhanced or impaired after MPK17 was knockdown or over-expressed, demonstrating that MPK17 is a novel MAPK element that has negative role in the *Xa21*-mediated immunity.

In rice-*Xoo* interactions, MAPKs have been shown to function as negative regulatory elements, such as OsMPK5 (Os03g17700) and OsMPK15 (Os11g17080) (Seo et al. 2011; Hong et al. 2019), positive regulatory elements, such as OsMPK7, OsMPK12 (Os06g49430, OsMPK12-1) (Seo et al. 2011; Xiao et al. 2017) or dual functional elements such as OsMPK6 (Shen et al. 2010). However, their roles in XA21-mediated resistance are unclear.

### MPK17 has Multiple Roles in Rice

Transcriptomic analysis revealed that the expression level of *OsMPK17* was down-regulated both in compatible and incompatible responses when inoculated with *Magnaporthe grisea* (Kumar et al. 2021). The abundance transcript levels of *OsMAPK17* was decreased under chilling condition at germination stage (Viana et al. 2021). In this study, dramatic alterations in a number of agronomic traits were observed when MPK17 was regulated, indicating that *OsMPK17* plays roles in rice development.

### MPK17 Regulates Transcriptional Factor WRKY62 Positively in the *Xa21*-Mediated Immunity

The downstream of MAPK cascade signaling is often transcription factors (Chen et al. 2021a, b). Five WRKY transcription factors (OsWRKY42 (Wang et al. 2021), OsWRKY62 (Peng et al. 2008), OsWRKY67 (Vo et al. 2017), OsWRKY68 (Zhu et al. 2021) and OsWRKY76 (Seo et al. 2011)) have been identified in the *Xa21*-mediated disease resistance pathway via genetic approach. It has been demonstrated that OsWRKY62 is localized in the nucleus, and over-expression of OsWRKY62 impairs resistance against *Xoo* (Peng et al. 2008). Bimolecular fluorescence complementation experiments showed that the intracellular kinase domain of XA21 protein and OsWRKY62 interact in the nucleus of rice protoplasts (Park and Ronald 2012). In Kitaake rice containing *Xa21*, RNAi and overexpression experiments demonstrated that both OsWRKY62 and OsWRKY76 are negative regulators for the *Xa21*-mediated resistance (Peng et al. 2008; Seo et al. 2011), while OsWRKY67 is a positive regulator (Vo et al. 2017).

In this study, in the background of *Xa21*, among the five above-mentioned WRKY transcription factors tested, WRKY62 was the only transcription factor whose abundance was positively correlated with MPK17 level after inoculated with *Xoo,* suggesting that the *MPK17* is likely the regulator of WRKY62 in *Xa21*-mediated resistance pathway.

### MPK17 Might be the Upstream Regulator of PR1A in *Xa21*-Mediated Immunity

In the process of plant-pathogen interactions, PRs play defensive functions to limit the reproduction of pathogens in plants. OsMPK15-mediated enhancement of resistance was correlated to the up-regulation of *PR1* and *PR10a* (Hong et al. 2019). In the OsMPK7 over-expression transgenic plants, the transcript levels of *PR1b* and *PR10* were significantly up-regulated after inoculated with *Xoo*, leading to enhanced resistance to bacterial blight (Jalmi and Sinha 2016). In the OsMPK4 (Os10g38950)-OX transgenic plants (*OsMPK4* was named *OsMPK6* in Shen (Shen et al. 2010)), the transcript level of *OsPR10a* was up-regulated after *Xoo* inoculation, indicating that OsMPK4 can regulated *OsPR10a* and contributes to bacterial blight resistance (Li et al. 2021).

The WRKY transcription factors may regulate the expression of PRs via binding to the W-box motif in the promoter region of the PR genes. In the background of *Xa21*, the transcriptional levels of *PR1a* and *PR10a* were down-regulated in the WRKY62-OX lines (Peng et al. 2008). Our study showed that the expression of PR1A is also associated with multiple factors, including time post *Xoo* inoculation or the number of *Xoo* cells, the existence of *Xa21* gene and regulation of MPK17 protein. The abundance of PR1A alone may not related with the resistance phenotype directly. It was demonstrated that WRKY42 binds to the W-box motif in the promoter region of *PR1a* and *PR1b* (Miao et al. 2014), and WRKY68 binds to the W-box motif in the promoter region of *PR1b* and *PR10a* (Yang et al. 2016). In this study, PR1A was found upregulated in both MPK17 knockdown- and overexpressing-plants when infected by *Xoo*, suggesting that MPK17 might be an upstream regulator of PR1A in the *Xa21*-mediated immunity.

### A Model in the Mid- and Late-Stages of *Xa21*-Mediated Immunity

**Fig. 11:**
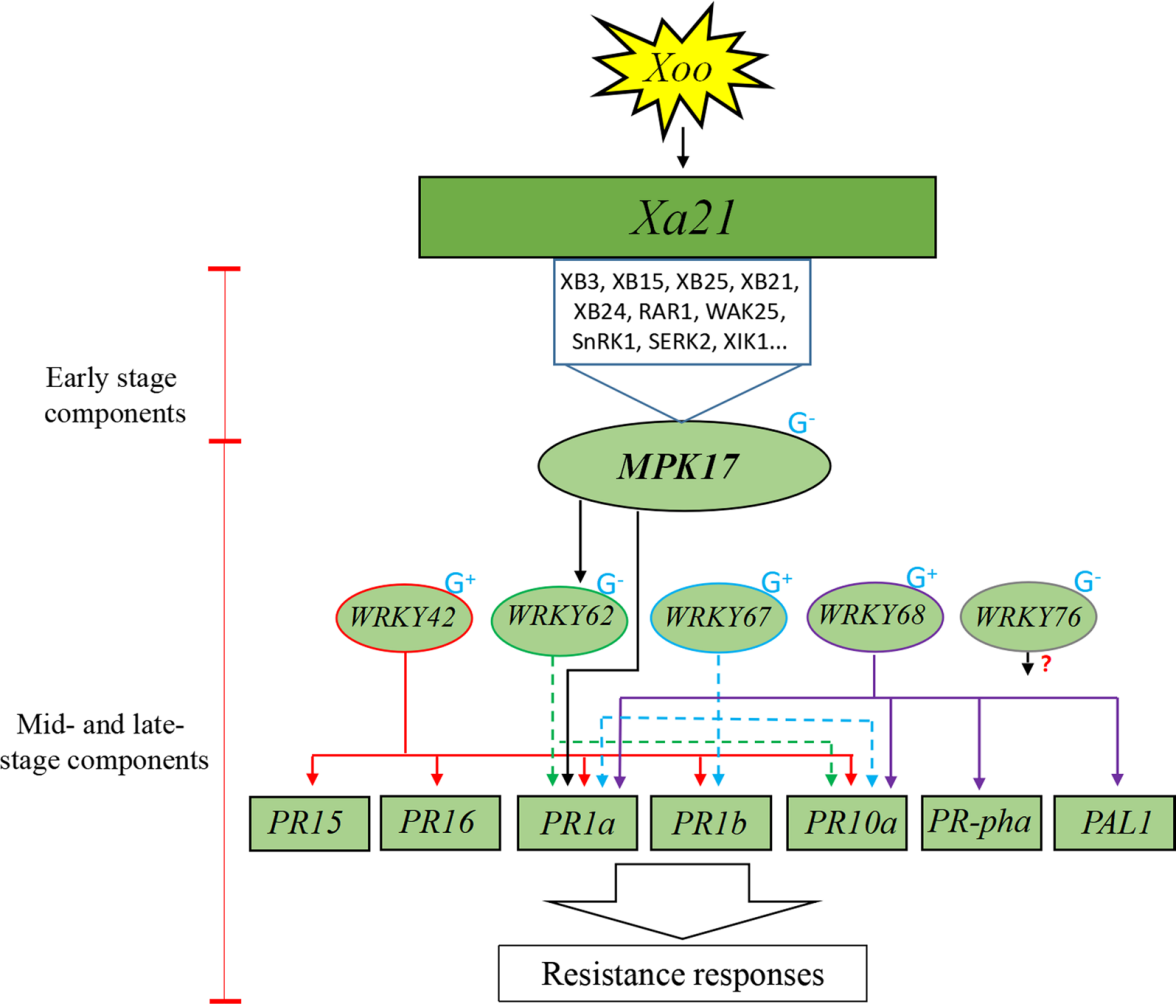
A model for the mid- and late-stages of the *Xa21*-mediated immunity. G^+^ and G^−^ denote the genetic evidence that supports a positive or negative regulator, respectively; solid and dotted lines denote WB or RT-PCR evidence, respectively

Over the past two decades, more than a dozen of components have been identified in *Xa21*-mediated immunity, which led to the proposal of a number of models for the immune system (Park et al. 2010b; Jiang et al. 2020; Kumar et al. 2020). To date, most components in *Xa21*-mediated immunity have been identified by yeast-two-hybrid or pull-down assays and can be defined as early-stage components. Based on the models for receptor kinase function in animal, the MAPK cascade is regarded as a core of stress responsive signaling (Chen et al. 2021a, b). In plant, the MAPKs activate downstream transcription factors and induce the expression of PRs leading to the defense response (Jalmi et al. 2016). The processes involved in the MAPK cascade and downstream components occur likely later in the defense response. To integrate MPK17 into the XA21-mediated immunity, we propose a two-stage model based on the data from our work and the studies by others (Fig. 11). The core of this model is the MPK17-WRKY62-PR1A axis, where MPK17 is the only *MAPK* gene identified with genetic evidence that supports its function in *Xa21*-mediated immunity. Additionally, five WRKY transcriptional factor genes (WRKY42 (Wang et al. 2021), WRKY62(Peng et al. 2008), WRKY67 (Vo et al. 2017), WRKY68 (Zhu et al. 2021) and WRKY76 (Seo et al. 2011)) are functional elements, and multiple pathogenesis-related genes (*PR15* (Wang et al. 2021), *PR16* (Wang et al. 2021), *PR1a* (Peng et al. 2008; Vo et al. 2017; Wang et al. 2021; Zhu et al. 2021), *PR1b* (Vo et al. 2017; Wang et al. 2021), *PR10a* (Peng et al. 2008; Vo et al. 2017; Wang et al. 2021; Zhu et al. 2021)), *PR-pha* (Zhu et al. 2021) and *PAL1* (Zhu et al. 2021)) play various defense roles in the pathway were supported by either WB or RT-PCR evidences. The identification of MPK17 provide a link between *Xa21* and downstream factors, however, the mechanisms underlying the interactions between MPK17 and these transcriptional factors and PRs warrant further investigation.

## Conclusions

In this study, it was found that the abundance of MPK17 is decreased in *Xa21*-mediated resistance. Transgenic MPK17-RNAi and MPK17-OX lines were produced in rice 4021 with *Xa21*. Compared with 4021, the resistance was enhanced in the MPK17-RNAi lines but impaired in the MPK17-OX lines, indicating that the *MPK17* gene plays a negative role in the *Xa21*-mediated immunity. Meanwhile, the MPK17-regulated rice plants showed altered agronomic traits, indicating that MPK17 also plays roles in the growth and development. Furthermore, in the MPK17-regulated transgenic rice plants, the expression of transcription factor WRKY62 and pathogenesis-related proteins PR1A was changed, suggesting that MPK17 may regulate the *WRKY62* and *PR1A* genes. A functional model along the *Xa21*-MPK17-WRKY62-PR1A pathway in the *Xa21*-mediated immunity is proposed.

## Supplementary Information

**Additional file 1**. **Fig. S1A**. Expression of MPK17 in rice TP309 leaves at different time points following inoculation with *Xoo*. **Fig. S1B**. Expression of XA21 in MPK1-RNAi and MPK17-OX transgenic rice plants. **Fig. S2**. Construction and verification of MPK17-RNAi transformation vector. **Fig. S3**. Identification of MPK17-RNAi transgenic lines by PCR and WB. **Fig. S4**. Identification of MPK17-OX in transgenic rice. **Fig. S5**. RT-PCR analysis of MPK17-RNAi and MPK17-OX transgenic rice plants. **Fig. S6**. *Xa21*-mediated resistance in rice at 31℃following overexpression of MPK17. **Fig. S7**. Effects of MPK17 abundance on the propagation of *Xoo* in rice leaves. **Fig. S8**. Expression of WRKY protein in rice leaves infected with *Xoo*. **Fig. S9**. Expression of pathogenesis-related protein PR1A in MPK17-regulated rice plants.

## Data Availability

All data generated or analyzed during this study are included in this published article or its supplementary information files or are available from the corresponding authors on reasonable request.

## Abbreviations

*Xoo*: *Xanthomonas oryzae* Pv*.oryzae*
PAMP: Pathogen-associated molecular pattern
PTI: PAMP-triggered immunity
ETI: Effector-triggered immunity
RLK: Receptor-like kinase
LRR: Leucine-rich repeat
MAPK: Mitogen-activated protein kinase
