# Supplementary material for: Rice MPK17 Plays a Negative Role in the Xa21-Mediated Resistance Against Xanthomonas oryzae pv. oryzae
Source: Rice (N Y). 2022 Aug 3;15:41. doi: 10.1186/s12284-022-00590-4 (PMC9349333; doi:10.1186/s12284-022-00590-4)
Supplement: Supplementary file 1 — Additional file 1. Fig. S1A. Expression of MPK17 in rice TP309 leaves at different time points following inoculation with Xoo. Fig. S1B. Expression of XA21 in MPK1-RNAi and MPK17-OX transgenic rice plants. Fig. S2. Construction and verification of MPK17-RNAi transformation vector. Fig. S3. Identification of MPK17-RNAi transgenic lines by PCR and WB. Fig. S4. Identification of MPK17-OX in transgenic rice. Fig. S5. RT-PCR analysis of MPK17-RNAi and MPK17-OX transgenic rice plants. Fig. S6. Xa21-mediated resistance in rice at 31℃following overexpression of MPK17. Fig. S7. Effects of MPK17 abundance on the propagation of Xoo in rice leaves. Fig. S8. Expression of WRKY protein in rice leaves infected with Xoo. Fig. S9. Expression of pathogenesis-related protein PR1A in MPK17-regulated rice plants. [file 12284_2022_590_MOESM1_ESM.docx]

**Supplementary information**

**Additional file**:

**Fig. S1A.** Expression of MPK17 in rice leaves of TP309 at different time points following inoculation with *Xoo*.

**Fig. S1B.** Expression of XA21 in MPK1-RNAi and MPK17-OX transgenic rice plants.

**Fig. S2.** Construction and verification of MPK17-RNAi transformation vector.

**Fig. S3.** Identification of MPK17-RNAi transgenic lines by PCR and WB.

**Fig. S4**. Identification of MPK17-OX in transgenic rice

Fig. S5. RT-PCR analysis of MPK17-RNAi and MPK17-OX transgenic rice plants

**Fig. S6**. Xa21-mediated resistance in rice at 31℃ following overexpression of MPK17.

**Fig. S7.** Effects of MPK17 abundance on the propagation of *Xoo* in rice leaves.

**Fig. S8.** Expression of WRKY protein in rice leaves infected with *Xoo*.

**Fig. S9.** Expression of pathogenesis-related protein PR1A in MPK17-regulated rice plants.


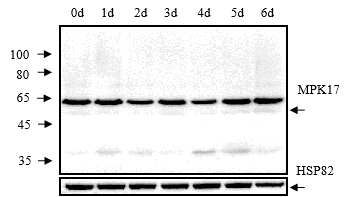


**Fig. S1A. Expression of MPK17 in rice leaves of TP309 at different time points following inoculation with Xoo**

Upper panel: Western blot detection of MPK17 protein. MPK17: anti-MPK17 antibody-detected band. Lower panel: anti-HSP82 antibody detected-band used as loading control.


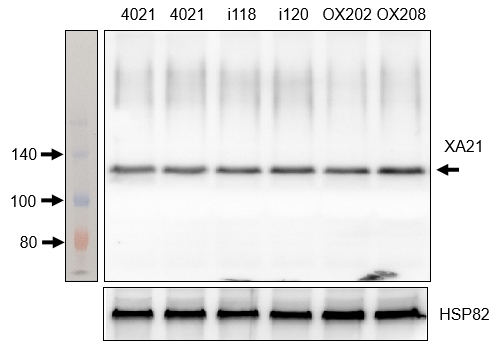


**Fig. S1B. Expression of XA21 in MPK1-RNAi and MPK17-OX transgenic rice plants.**

Upper panel: Western blot detection of XA21 protein using anti-cMYC antibody. Lower panel: anti-HSP82 antibody detected-band used as loading control.

4021: TP309 transgenic line harboring the *Xa21* gene. i118, i120: independent MPK17-RNAi transgenic lines; OX202 and OX208: independent MPK17-OX transgenic lines.


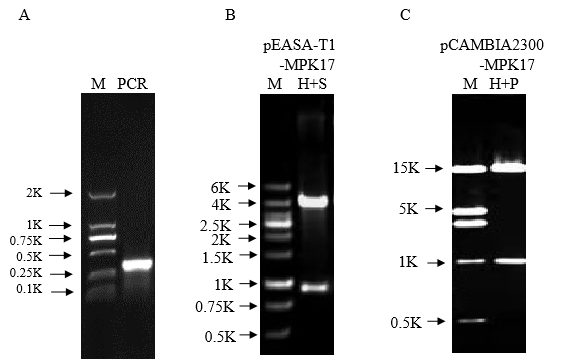


**Fig. S2. Construction and verification of MPK17-RNAi transformation vector.**

(A) PCR products used to construct of MPK17-RNAi.

(B) Verification of MPK17-RNAi fragment inserted into the intermediate vector.

(C) Enzyme digestion verification of the transformation plasmid pCAMBIA2300-MPK17-RNAi.

M: Molecular weight marker; PCR: MPK17-RNAi fragment; pEASA-T1-MPK17: MPK17 RNA interference fragment was integrated into intermediate vector pEASA-T1; pCAMBIA2300-MPK17: MPK17 RNA interference fragment was integrated into pCAMBIA2300 plasmid. H: *Hin*d Ⅲ; S: *Sac*Ⅰ; P: *Pst* Ⅰ.


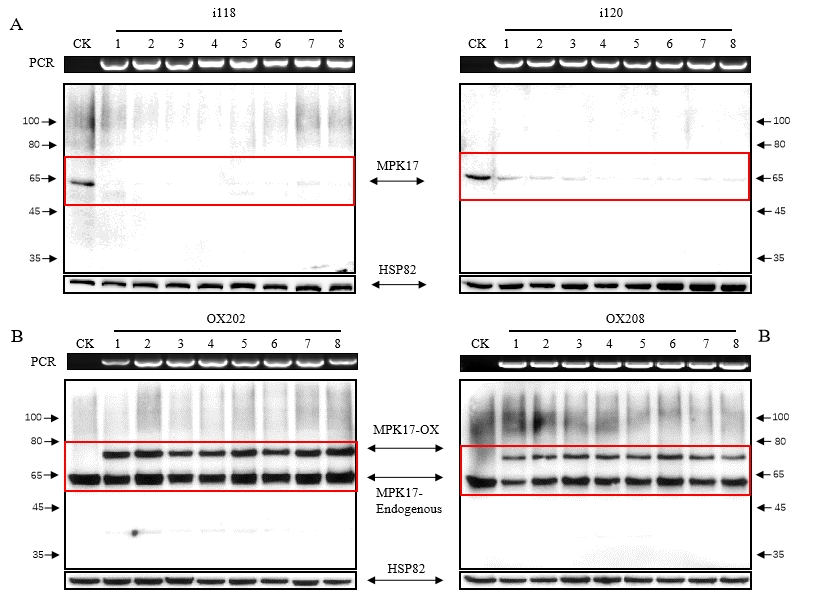


**Fig. S3. Identification of MPK17-RNAi and overexpression transgenic lines by PCR and WB.**

1. **Identification of MPK17-RNAi transgenic lines by PCR and WB.**
2. **Identification of MPK17-OX transgenic lines by PCR and WB.**

The red frames in A indicate the portion showed in Fig. 2 and the red frame in B indicated the protein showed in Fig. 4, see illustrations in Fig. 2 and Fig. 4 for detail.


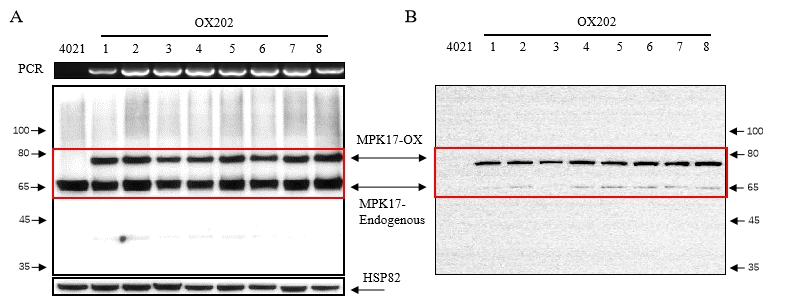


**Fig. S4. Identification of MPK17-OX in transgenic rice**

At seedling stage, leaf tissues were collected from the recipient 4021 and 8 transgenic plants from OX202 line with over-expressed MPK17. PCR: PCR genotyping results done with primers (5’-GCGGTACCATGGGCGGCCGCGCCCGCTC-3’ and 5’-GCGAGCTCGGTTTTCAGTTGAGCAAC-3’). Total proteins were isolated and separated by SDS-PAGE. Anti-MPK17 antibody were used to detect MPK17 protein on PVDF membrane (A). Anti-MPK17 antibody was washed off and anti-HA antibody was applied to detect signal of MPK17 (B).

The red frames indicate the portion showed in Fig. 2, see illustrations in Fig. 2 for detail.


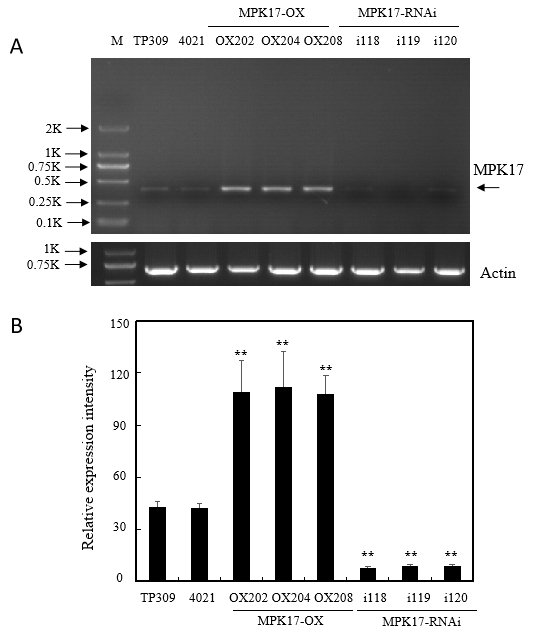


Fig. S5. RT-PCR analysis of MPK17-RNAi and MPK17-OX transgenic rice plants

Seeds of TP309, 4021, three independent lines of MPK17-OX (OX202, OX204 and OX208) and three independent MPK17-RNAi lines (i118, i119 and i120) were soaked for three days at 37℃and germinated for 7days in incubator (30 ℃, humidity 50~60%, 15 h light/9 h dark cycle, light intensity 4100 Lux). Seedlings were harvested to isolate total RNA using TransZol Up RNA Kit (Quanshijin Co., Ltd. China). HiFiScript kit (Kangwei Co., Ltd. Beijing, China) was used to eliminate genomic DNA and reverse transcription was carried out to prepare template cDNA.

The upstream primer for PCR amplification of MPK17 gene was 5’-ATGAGGAAAAAGCCTCGGGTTCC-3’. The downstream primer was 5’-TCACCCTTTGAAACTCCTTCCTCC -3’. The upstream primer for PCR amplification of actin gene (Os03g50885) was 5’-CCTCGTCTCGACCTTGCTGGG-3’, the downstream primer was 5’-GAGAACAAGCAGGAGGACGGC-3’. PCR amplifications were carried out using the follow cycling parameters for both MPK17 and actin gene: pre-denaturation at 94 ℃ for 3 min, followed by 40 cycles of denaturation at 94 ℃for 45 s, annealing at 56 ℃ for 45 s, and extension at 72 ℃ for 90 s, with a final extension at 72 ℃ for 10 min. The PCR products were separated by electrophoresis using 1.0 % agarose gel (A). The RT-PCR experiments were repeated for three times. Image-Pro Plus 6.0 software were used to extract the signal intensity of MPK17 PCR band. The relative intensities of RNAi and OX lines were compared with controls (TP309 and 4021) respectively (B). **designate difference at p<0.01 level.


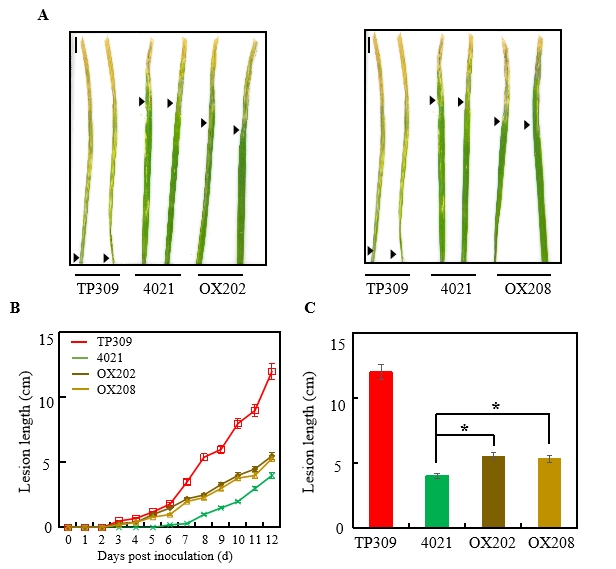


**Fig. S6. Xa21-mediated resistance in rice at 31℃ following overexpression of MPK17.**

(A) Lesions in inoculated leaves of OX202, OX208 and control plants at 12 dpi.

(B) Lesion length at different dpi.

(C) Lesion length at 12 dpi.

Arrows indicate the frontline of lesions. * and **designate difference at p<0.05 and p<0.01 levels respectively. Bar = 1 cm.

OX202 and OX208: independent MPK17-OX transgenic lines; TP309: Japonica rice variety; 4021: TP309 transgenic line harboring the *Xa21* gene.


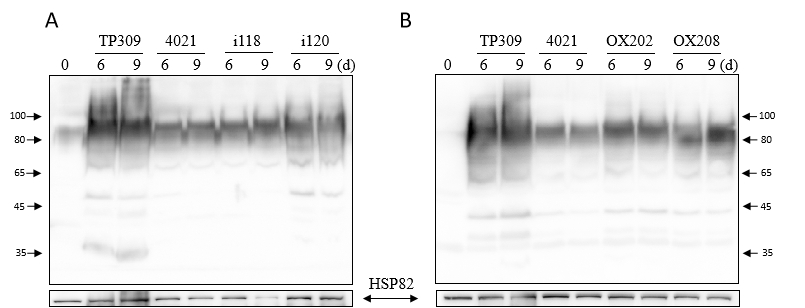


**Fig. S7. Effects of MPK17 abundance on the propagation of *Xoo* in rice leaves.**

(A) Detection of *Xoo* protein in leaves of MPK17-RNAi transgenic rice.

(B) Detection of *Xoo* protein in leaves of MPK17-OX transgenic rice.

Inoculated rice plants were cultivated at 31℃ (MPK17-RNAi transgenic plants) or 27℃ (MPK17-OX transgenic plants). The whole leaves were collected at 0, 6, 9 days, total proteins were isolated and separated by SDS-PAGE, then detected by anti-*Xoo* antibody.

HSP82: anti-HSP82 antibody detected band used as loading control. i118, i120: independent MPK17-RNAi transgenic lines; OX202, OX208: independent MPK17-OX transgenic lines; TP309: Japonica rice variety; 4021: transgenic TP309 line harboring the *Xa21* gene.


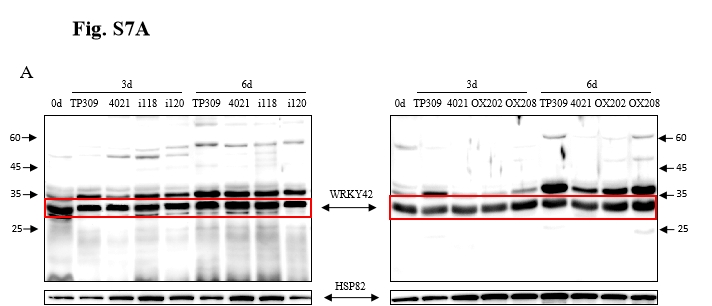


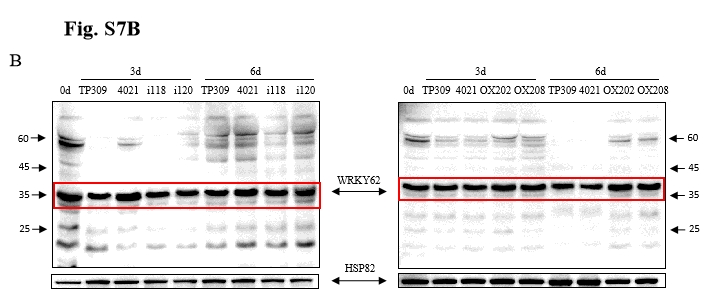


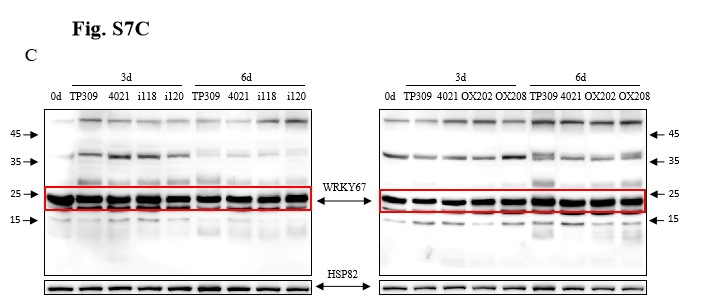


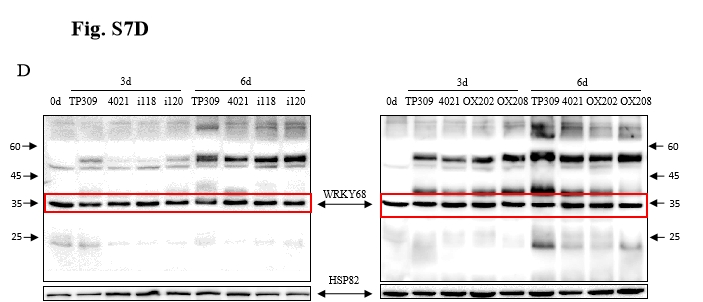


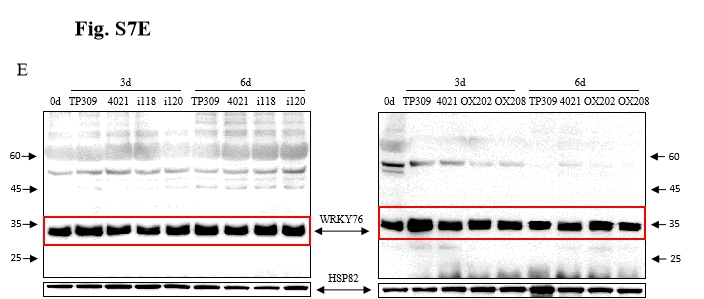


**Fig. S8. Expression of WRKY protein in rice leaves infected with *Xoo*.**

**Fig. S8A:** WB detection of the expression of WRKY42 protein.

**Fig. S8B:** WB detection of the expression of WRKY62 protein.

**Fig. S8C**: WB detection of the expression of WRKY67 protein.

**Fig. S8D:** WB detection of the expression of WRKY68 protein.

**Fig. S8E:** WB detection of the expression of WRKY76 protein.

Total protein were isolated from inoculated leaves ( ±1cm within lesion line) of 3d or 6d and resolved by SDS-PAGE or Tricine-SDS-PAGE. Then detected by anti-WRKY protein antibodies. HSP82: anti-HSP82 antibody detected band used as loading control. i118, i120: independent MPK17-RNAi transgenic lines; OX202, OX208: independent MPK17-OX transgenic lines; TP309: Japonica rice variety; 4021: transgenic TP309 line harboring the *Xa21* gene.

The red frames indicate the portion demonstrated in Fig. 9.


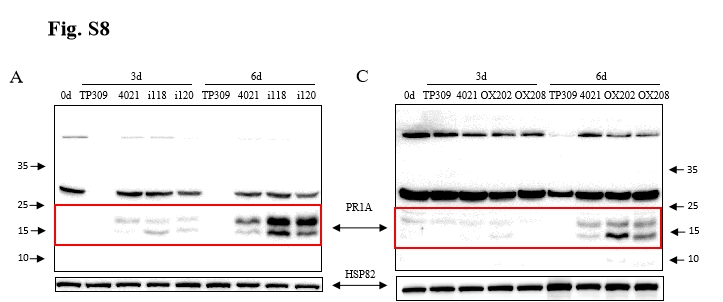


**Fig. S9. Expression of PR1A protein in MPK17-regulated rice plants.**

Total protein were isolated from inoculated leaves ( ±1cm within lesion line) at 0, 3, and 6 dpi and resolved by SDS-PAGE. WB detection were carried out using anti-PR1Aantibody. i118, i120: independent MPK17-RNAi transgenic lines; OX202, OX208: independent MPK17-OX transgenic lines; TP309: Japonica rice variety; 4021: transgenic TP309 line harboring the *Xa21* gene.

The portion in red box were showed in in Fig. 10.
